# Design and Characterization of Ricin Based Immunotoxins Against EPHA2 Receptor for Breast Cancer Therapy: An In-Silico Study

**DOI:** 10.5812/ijpr-151574

**Published:** 2025-02-22

**Authors:** Atefeh Faraz, Jafar Amani, Sedigheh Arbabian, Shohreh Zare Karizi, Maryam Bikhof Torbati

**Affiliations:** 1 Department of Cellular and Molecular Biology, Faculty of Biological Science, North Tehran Branch, Islamic Azad University, Tehran, Iran; 2 Applied Microbiology Research Center, Systems Biology and Poisonings Institute, Baqiyatallah University of Medical Sciences, Tehran, Iran; 3 Department of Genetic and Biotechnology, Varamin Pishva Branch, Islamic Azad University, Varamin, Iran; 4 Department of Biology, Yadegar-e-Imam Khomeini (RAH) Shahr-e-Rey Branch, Islamic Azad University, Tehran, Iran

**Keywords:** Immunotoxin, Ricin, EPHA2 Receptor, Breast Cancer, Bioinformatics Analysis

## Abstract

**Background:**

One of the most promising strategies to combat cancer is the use of immunotoxins.

**Objectives:**

This study aimed to design two immunotoxins composed of antibody fragments against the EphA2 receptor, which is highly expressed in breast cancer.

**Methods:**

EphA2-N-ricin and EphA2-C-ricin were designed by fusing scFv against the EphA2 receptor with the A chain of ricin in varying orders. mFold was used to analyze the mRNA stability of the constructs. The 2D and 3D protein structures of the constructs were predicted using prediction tools and verified by quality assessment tools. The physicochemical properties were calculated using ProtParam. Docking between the constructs and the EphA2 receptor was performed using HADDOCK software, and the 2D interaction plots of the complexes were generated using LigPlus. A 100 ns molecular dynamics (MD) simulation was conducted for docked complexes using Gromacs. Ultimately, the allergenicity and antigenicity of the constructs were determined.

**Results:**

The designed immunotoxins had stable mRNAs, reliable 2D and 3D protein structures, and demonstrated high affinity and stable interactions with the receptor protein, as revealed by docking and MD analyses. Higher binding affinity and stability were observed for construct 2. Moreover, the designed immunotoxins lacked allergenicity and were identified as antigens.

**Conclusions:**

Based on these observations, it is reasonable to conclude that both designed immunotoxins could serve as suitable immunotoxins; however, construct 2 exhibits more promising properties. Given these results, these immunotoxins could be used in empirical studies to treat breast cancer in vitro or in vivo.

## 1. Background

Breast cancer is the most common fatal disease among women (1). In terms of mortality, it ranks as the fifth most common cancer, particularly for women (2). As the most prevalent malignancy in women, breast cancer has a prevalence rate ranging from 21.4% (3) to 32% (4). Given the high incidence of breast cancer, it is critical to investigate efficacious therapeutic approaches for its management. Despite significant advancements in cancer therapeutic research, breast cancer continues to have one of the highest prevalence and mortality rates.

Erythropoietin-producing hepatocellular (EPH) carcinoma receptors are important for normal cellular function, especially in epithelial cells. They are the most significant class of receptor tyrosine kinases (RTKs) (5). In 1987, EphA1, the first EPH receptor ever defined, was discovered in liver cancer cells via an RTK screening (6). The EPH receptor signaling aids a variety of biological processes, most of which result in cell-cell adhesion or repulsion. Thus, EPH receptors and their ligands play crucial roles in blood vessel formation, neuronal targeting, and tissue patterning in the embryo (7, 8). However, Eph proteins are overexpressed in many cancers and are present in high quantities in these diseases (6). The expression level of the EphA2 receptor, which belongs to the EPH receptor family, increases in breast cancer cells (9), making it an ideal candidate for targeted breast cancer therapy (10).

Traditional chemotherapy methods work on the premise that cancer cells have a faster growth rate. However, chemotherapy agents target almost all cells, including normal or non-cancerous cells, which can result in toxicity and various side effects (11). Among current treatments, targeted therapies have shown great promise in cancer treatment (10, 12). In this regard, numerous monoclonal antibodies (mAbs) have been produced as targeted therapies to target different receptors overexpressed in cancer cells. Immunotoxins are a relatively recent class of targeted therapy agents in cancer treatment (13). An immunotoxin specifically recognizes the cancer cell and then triggers cell death. An immunotoxin is composed of mAbs or a portion of them, which is genetically or chemically coupled with a toxic agent. The toxin part of an immunotoxin is derived from different sources, such as plant toxins (e.g., ricin) (14) or bacterial toxins (15) like diphtheria toxin or Pseudomonas exotoxin.

To design an immunotoxin, several points should be considered. Since experimental validation of immunotoxins is expensive and time-consuming, it is prudent to first investigate immunotoxin design and properties through bioinformatics studies (16). As any immunotoxin is composed of two parts—a toxin and an antibody—and this chimeric protein is a new biological feature, bioinformatics or in silico analyses can provide useful data to determine if this chimeric protein is suitable for development in laboratory settings. Important aspects such as physical and chemical properties, mRNA stability, secondary and tertiary structure stability, immunogenicity, and solubility should be analyzed through bioinformatics before experimental setup (17).

In this study, we use in silico analyses to investigate two chimeric immunotoxins (EphA2-C-ricin and EphA2-N-ricin) designed for breast cancer treatment. Ricin toxin is a type II ribosomal activity inhibitor and a byproduct of the castor bean (Ricinus communis) that can be ingested orally, injected intramuscularly, or inhaled, resulting in poisoning and death. Ricin has been categorized as a bio-threat agent and poses a significant risk to public safety due to its high toxicity, stability, and availability (18). There is currently no effective counteragent against ricin toxin. One effective strategy for preventing and treating ricin poisoning is neutralizing antibody therapy. Ricin has a molecular weight between 60 and 64 kDa and consists of 541 amino acids. The ricin holotoxin comprises Ricin toxin A chain (RTA) and Ricin toxin B chain (RTB), joined by a disulfide link. RTA, an N-glycosidase, can inactivate the ribosome by removing adenine from the 4324th ribonucleic acid on the 28S rRNA (19).

## 2. Objectives

Therefore, we generated ricin and EphA2-containing immunotoxins by attaching the N or C terminal of subunit A of ricin to antibody fragments (single chain variable fragment or scFv) against the EphA2 receptor. This approach allows for specific targeting of cancer cell antigens, addressing the specificity issue in traditional chemotherapy methods.

## 3. Methods

### 3.1. Design of Recombinant Immunotoxins

Two immunotoxins were constructed by tandem fusion of the subunit A of ricin (sequence retrieved from UniProt: P02879) with scFv against the EphA2 receptor. The sequence of the scFv was retrieved from the 1F12 mAb of Patent number US20090304721, which was designed using three repeats of the G4S linker in our previous study (10). SnapGene Viewer software (version 5.3.1) was used to analyze the correct construction of the sequences of the designed immunotoxins.

### 3.2. The Secondary and Tertiary Structure Prediction and Quality Assessment

The GOR secondary structure prediction method version IV and the AlphaFold2 server were used to predict the secondary and tertiary structures of constructs 1 and 2, respectively. AlphaFold is a protein 3D structure prediction algorithm that greatly outperformed other methods in the challenging 14th Critical Assessment of Protein Structure Prediction (CASP14) and demonstrated accuracy competitive with experimental structures in a majority of cases (20).

For quality assessment, the PDB format of the best-identified structural analog from the AlphaFold server for each immunotoxin was imported into the RAMACHANDRAN plot server (https://www.umassmed.edu/zlab/) to obtain the plot. Additionally, the QMEAN server was used to estimate the quality of the obtained 3D models. The ERRAT and VERIFY 3D tools were also used to assess the quality of predicted structures through the UCLA-DOE LAB SAVES v6.1.

### 3.3. Physical-Chemical Properties

ProtParam was used to compute various physical and chemical parameters of constructs 1 and 2, including molecular weight, theoretical pI, amino acid composition, atomic composition, extinction coefficient, estimated half-life, instability index, aliphatic index, and grand average of hydropathicity.

### 3.4. Protein-Receptor Docking

The HADDOCK Server v.24 (21) was used to investigate the potential interaction between the designed immunotoxins and their target receptor protein. In this context, the structure of EphA2, the target receptor, was obtained from the RCSB Protein Data Bank (PDB). The structures of the designed immunotoxins and EphA2 were prepared for docking analyses by removing non-protein atoms using the PDBEditor software (22). The prepared structures were then used as input files for the docking analyses in the HADDOCK Server. For the docking analyses, the scFv regions of the designed immunotoxins and the entire EphA2 protein were designated as the active amino acids involved in the interaction.

### 3.5. Docking Refinement and Binding Affinity Calculation

The structure of the best complexes between the designed immunotoxins and the EphA2 receptor was used as the input file for docking result refinement. The complexes were edited using FireDock. The FireDock web server performs high-throughput, flexible refinement and scoring of protein-protein docking solutions. The binding affinity between the designed immunotoxins and EphA2 was calculated using PRODIGY (PROtein binDIng enerGY prediction). This server provides a collection of web services focused on predicting binding affinity in biological complexes and identifying biological interfaces from crystallographic ones.

### 3.6. Visual Presentations

LigPlus software was utilized for the automatic generation of 2D interaction diagrams for the complexes of the designed immunotoxins and EphA2. The refined protein complexes were used to visualize the interactions between the two protein chains, highlighting the amino acids involved at the protein interfaces.

### 3.7. Molecular Dynamics Simulation

Protein structure simulations and the enhancement of 3D structures can be achieved through molecular dynamics (MD) simulation, which replicates structural alterations in biological molecules over a specified period. The WebGro server was used to conduct the MD simulation. The refined 3D structures of the docked complexes were simulated for 50 nanoseconds in water using molecular dynamics modeling. WebGro performs detailed computer simulations of molecular behavior in a liquid solution using the GROMACS tool. The default parameters commonly used for GROMACS simulations were applied.

The Steepest Descent integrator was employed to perform 5000 steps of energy minimization. NVT/NPT was selected as the equilibration type, with a temperature of 300 K and a pressure of 1 bar. The simulation was conducted in a Triclinic water box using the GROMOS96 43a1 force field, with NaCl (0.15 M) serving as the neutralizing salt.

### 3.8. The mRNA Structure Prediction

To predict the secondary structure of the mRNA expressed from the constructs and assess its stability, the RNA sequence was imported into the RNA folding form of the mFold web server. The energy dot plot and thermodynamic details were obtained for the chimeric gene constructs.

### 3.9. Allergenicity and Antigenicity

To assess the allergenicity and antigenicity potential of the chimeric protein, the AlgPred server and the VaxiJen server were used, respectively. AlgPred predicts allergens based on the similarity of known epitopes to any region of the protein. Additionally, its IgE epitope mapping feature allows users to identify the position of epitopes within the protein. VaxiJen is a server designed for alignment-independent prediction of protective antigens. It enables antigen classification based solely on the physicochemical properties of proteins, without relying on sequence alignment.

## 4. Results

### 4.1. Design and Generation of Recombinant Immunotoxins

**Figure 1.**
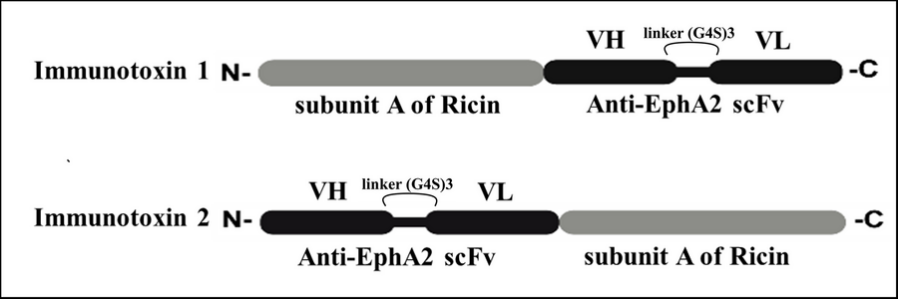
The schematic picture of construct 1 and 2. The light gray and black colors represent subunit A of ricin and scFv against the EphA2 receptor.

Two new recombinant chimeric immunotoxins were designed. In this context, from the N-terminal to the C-terminal, the fusion genes subunit A ricin–EphA2 (construct 1) and EphA2–subunit A ricin (construct 2) were created. Figure 1 illustrates the different orientations of the ricin and scFv fragments relative to EphA2 in construct 1 versus construct 2. Further analyses of these constructs were conducted using various bioinformatics approaches in the following sections.

### 4.2. The Prediction of Secondary and Tertiary Structure of Immunotoxins and Validity Assessment

The GOR secondary structure prediction method version IV and the AlphaFold server were used to predict the secondary and tertiary protein structures, respectively. The GORIV server predicted the presence of alpha helix, extended strand, and random coil structures in both constructs. The percentage of alpha helix, extended strand, and random coil structures was 22.46, 26.84, and 50.7% for construct 1 and 23.92, 26.16, and 49.91% for construct 2. 

**Figure 2.**
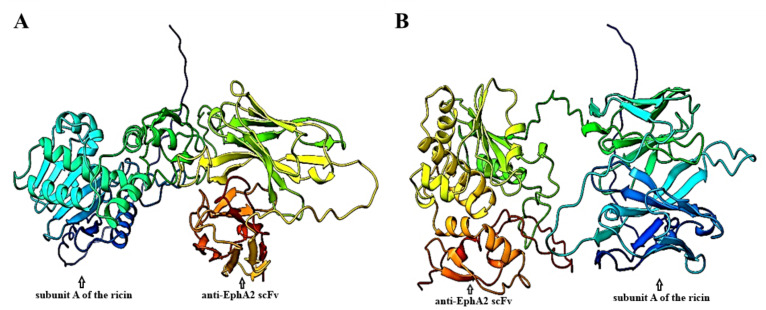
Tertiary protein structure prediction of recombinant immunotoxins by AlphaFold server. The structure of the best-ranked models is depicted for both A, construct 1; and B, construct 2.

**Figure 3.**
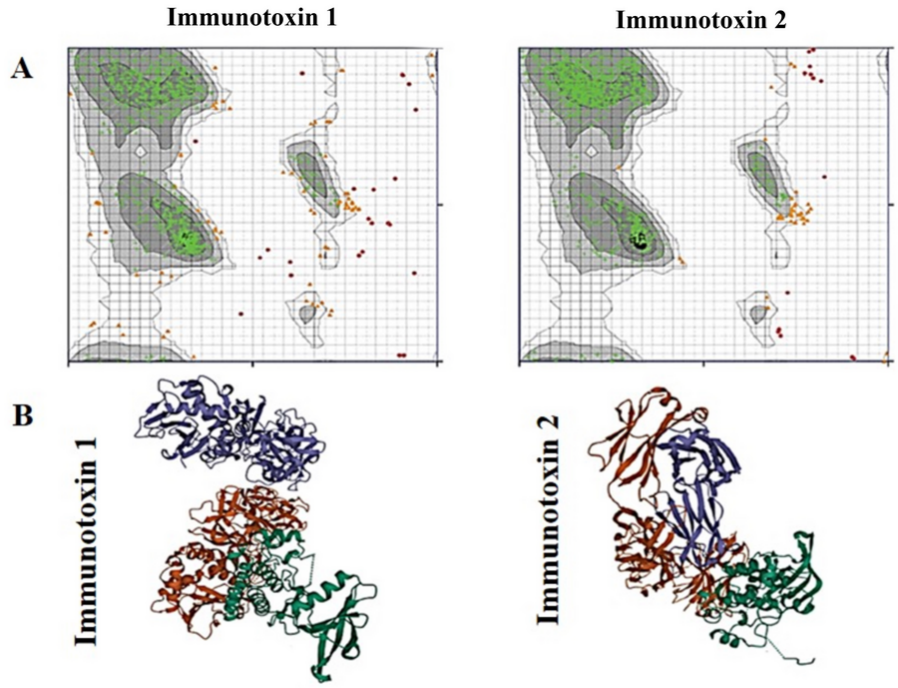
The Ramachandran plots and ligand-receptor docking of immunotoxins. A, the Ramachandran plots. Highly preferred, preferred, and questionable observations are shown as green crosses, brown triangles, and red circles, respectively; B, ligand-receptor docking. The EphA2 receptor is in blue, the subunit A of the ricin is in green, and the anti-EphA2 scFv is in red.

The AlphaFold server successfully predicted five top models for the tertiary structures of both constructs. The predicted models are shown in Figure 2. For quality assessment, Ramachandran plots were generated using the RAMACHANDRAN plot server. As shown in Figure 3A, proteins expressed from both construct 1 and construct 2 exhibited acceptable conformations. According to the obtained results, 81.2% and 82.2% of the amino acids from construct 1 and construct 2, respectively, were found in the favored regions, while only 4.2% and 1.6% of the amino acids from construct 1 and construct 2, respectively, were located in the disallowed regions. 

Additionally, QMEAN scores of 0.74 were assigned to both construct 1 and construct 2. The ERRAT results indicated that construct 1 and construct 2 had an Overall Quality Factor of 86.5731 and 89.4212, respectively. VERIFY 3D results demonstrated that 73.84% of the residues in construct 1 had an averaged 3D-1D score of ≥ 0.1, while construct 2 also had 73.84% of residues within the same threshold (Appendix 1 in Supplementary File). These results suggest that the predicted 3D structures exhibit moderate quality.

### 4.3. Physical-Chemical Properties of Immunotoxins

Based on the ProtParam software, the physicochemical properties of both immunotoxins were calculated. The protein expressed from construct 1 consists of 570 amino acids with a molecular weight of 62,246.31 Da. Its theoretical pI is 9.07, with 39 negatively charged residues (Asp + Glu) and 47 positively charged residues (Arg + Lys). The extinction coefficient is 77,615 M⁻¹ cm⁻¹. The estimated half-life is 30 hours in mammalian reticulocytes (in vitro), more than 20 hours in yeast (in vivo), and more than 10 hours in Escherichia coli (in vivo). Its instability index is computed to be 39.33, indicating that it is a stable protein. 

The protein expressed from construct 2 consists of 581 amino acids with a molecular weight of 63,524.67 Da. Its theoretical pI is 8.97, with 40 negatively charged residues (Asp + Glu) and 47 positively charged residues (Arg + Lys). The extinction coefficient and estimated half-life are identical to those of the protein expressed from construct 1. Its instability index is computed to be 38.65, also indicating that it is a stable protein (Appendix 2 in Supplementary File).

### 4.4. Protein-Receptor Docking of Immunotoxins

The structure of the EphA2 receptor was obtained from the RCSB PDB under the ID 5NKA. After the protein preparation step, the predicted 3D structure of each designed immunotoxin was docked with the 3D structure of EphA2, extracted from 5NKA. The results of the docking analyses indicated that the designed immunotoxins can interact with the EphA2 receptor through their scFv region. 

The docking analysis results, including the complexes of immunotoxin structures in interaction with the EphA2 structure, were stored as PDB files and visualized using the 3D view of Mol software (Figure 3B). The HADDOCK score for the best-docked complex between construct 1 and EphA2 was 914.6 ± 47.0, while the complex between construct 2 and EphA2 had a HADDOCK score of 926.1 ± 47.1. These results suggest that the docking between construct 2 and EphA2 was more favorable than the docking between construct 1 and EphA2.

### 4.5. Interaction Refinement and Binding Affinity Analyses

The best complexes between the 3D structure of the immunotoxin and EphA2 were refined using the FireDock server. The refined complexes were then used to calculate the binding affinity between the immunotoxin and EphA2 structures. The binding affinity between construct 2 and EphA2 was -20.9 kcal/mol (Kd: 4.6e-16 M), while the binding affinity between construct 1 and EphA2 was calculated to be -19.1 kcal/mol (Kd: 9.2e-15 M) (Appendix 3 in Supplementary File). The obtained results indicate that the interaction between construct 2 and EphA2 is more stable compared to the interaction between construct 1 and EphA2.

### 4.6. 2D Protein Interaction Plot

The amino acids involved in the interaction between construct 1, construct 2, and EphA2 were mapped at the protein interfaces of the docked protein complexes. Additionally, the type of each bond between the amino acids at the protein interfaces was identified using the LigPlus software. Hydrogen bonds and hydrophobic interactions were the most prevalent interaction types between the designed constructs and the EphA2 receptor. A higher number of these interactions indicates a stronger binding affinity between the interacting molecules. 

**Figure 4.**
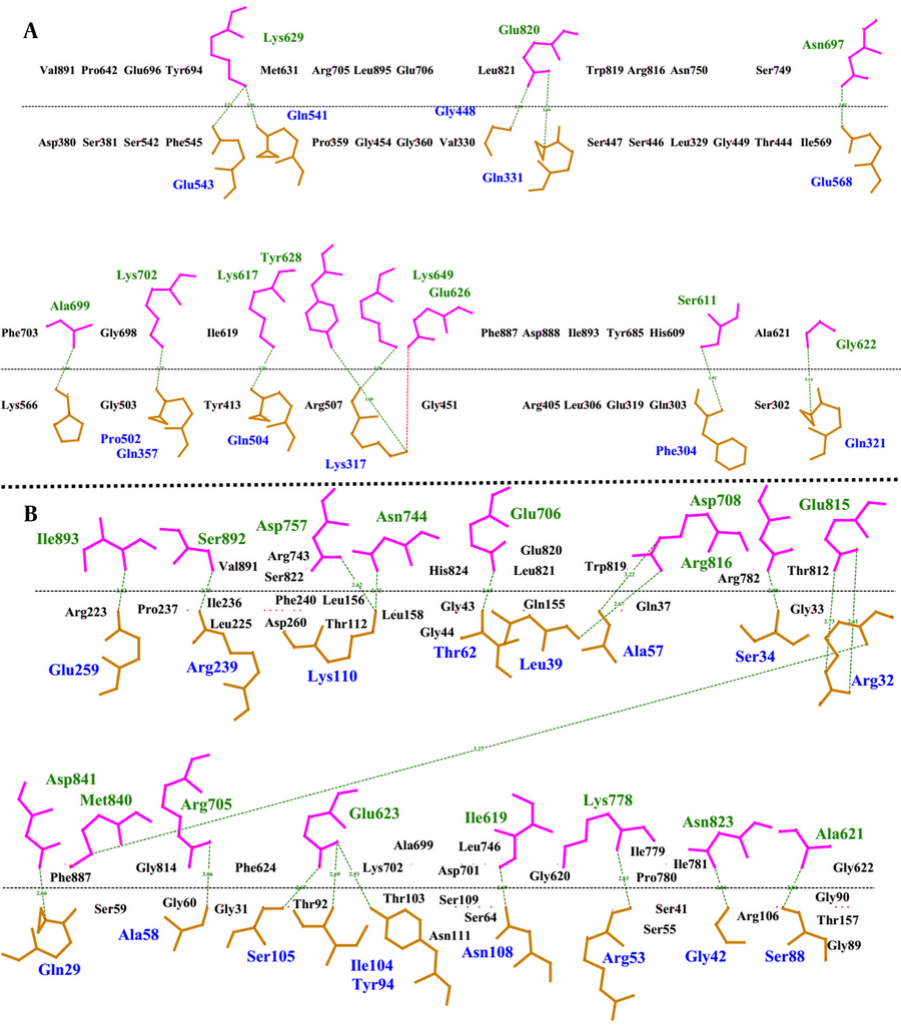
2D interaction plot for A, construct 1 and EphA2; and B, construct 2 and EphA2. The hydrogen bonds are shown in green and the amino acids written in black are involved in hydrophobic interactions.

In this context, the analysis of the bond types and numbers at the interfaces of the docked complexes revealed that the interaction between construct 2 and EphA2 involves a greater number of both interaction types, making it more stable compared to the interaction between construct 1 and EphA2. For example, construct 2 and EphA2 formed 20 hydrogen bonds, whereas construct 1 and EphA2 formed 12 hydrogen bonds (Figure 4). Similarly, the number of hydrophobic interactions was also higher in the complex of construct 2 and EphA2.

### 4.7. Molecular Dynamics Simulation

To analyze the behavior of the docked structures of construct 1 and construct 2 in complex with the EphA2 receptor at an atomic level and to study their dynamics over time, MD simulations were performed. The MD simulation demonstrated that the selected docked structures of construct 1 and construct 2 in complex with EphA2 reached equilibrium and a stabilized condition after 50 ns of MD. 

**Figure 5.**
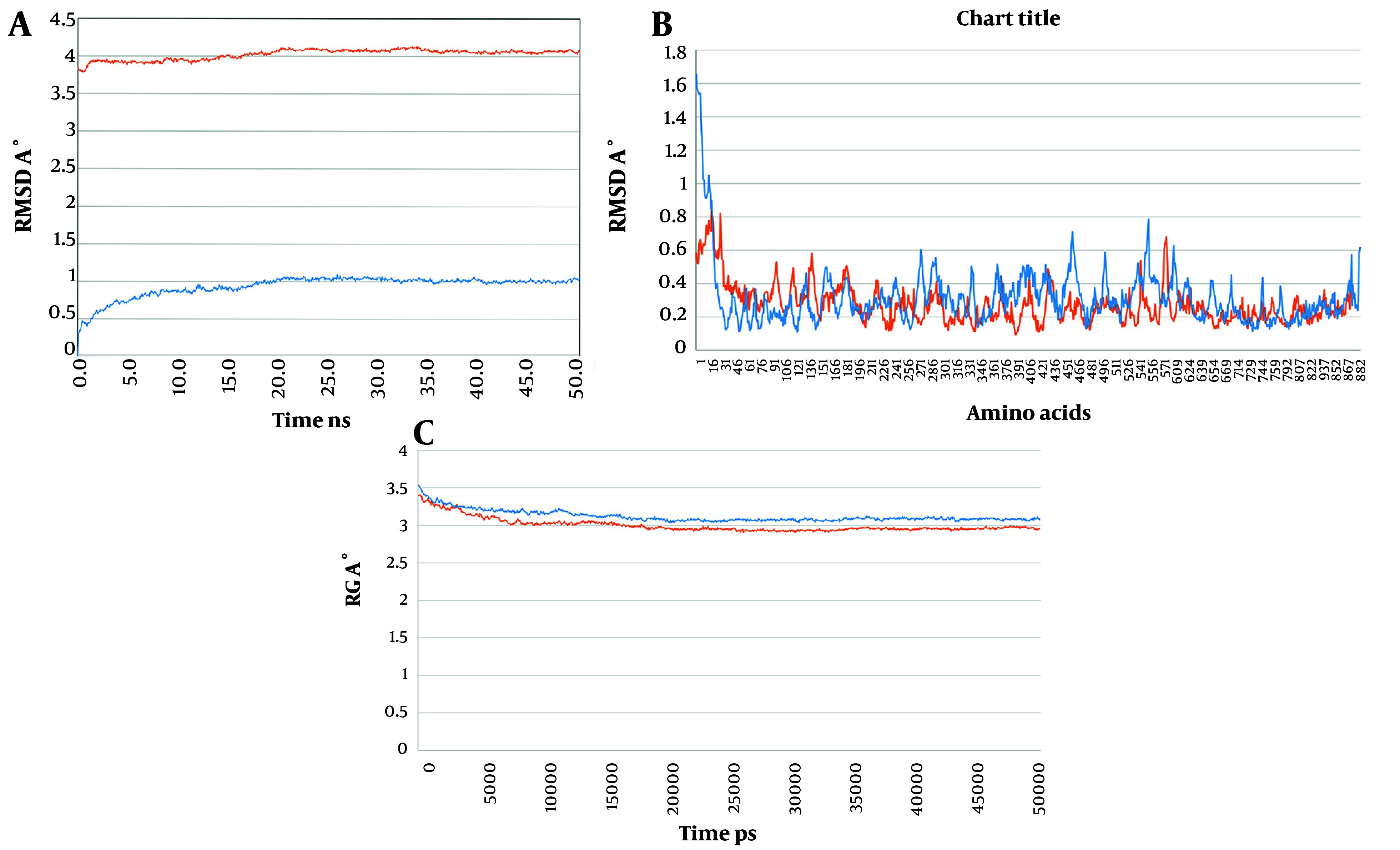
Results of MD simulation. A, RMSD variation during the MD simulation for the complex of construct 1 and construct 2 with the EphA2; B, RMSF variation during the MD simulation for the complex of construct 1 and construct 2 with the EphA2; C, Rg variation during the MD simulation for the complex of construct 1 and construct 2 with the EphA2. The plots for construct 1 is in orange and the plots for the construct 2 is in blue).

The root mean square deviation (RMSD) method was used to calculate the average distance between the frame-by-frame simulated structures and the reference structure's backbone atoms. The backbone RMSD plot of the docked complexes showed that the structures achieved a stable state after 20 ns of MD simulation, with a decrease in RMSD as the simulation progressed (Figure 5). This suggests that the structures initially underwent conformational adjustments before stabilizing over time. 

The radius of gyration (Rg) was calculated to assess the radii of gyration along the x, y, and z axes, as well as the structural compactness of the molecules over time. The results indicated that during the simulation, the docked structures maintained their structural compactness and stability, with no significant unfolding or expansion. 

Additionally, the root-mean-square fluctuation (RMSF) plot was generated to assess the positional deviation of the structures relative to a reference structure. This plot revealed the flexibility of amino acids during the simulation. The obtained results showed that the complex of construct 2 with EphA2 exhibited lower positional deviation for the amino acids, suggesting that construct 2 adopts a more rigid conformation compared to construct 1. These findings indicate that both docked complexes stabilized over time, with construct 2 demonstrating greater stability and reduced flexibility, potentially reflecting stronger binding interactions with EphA2.

### 4.8. The Prediction of the mRNA Structure

The mFold software predicted 47 and 46 secondary structures (circular structure plots) for the mRNAs expressed from constructs 1 and 2, respectively. These predicted structures represent a comprehensive range of possible mRNA conformations that can form under the given conditions. The range of initial ΔG (Gibbs free energy) values was between -663.00 and -635.30 kcal/mol for construct 1 and between -671.50 and -642.80 kcal/mol for construct 2. 

The ΔG values indicate the stability of the secondary structures, with more negative values correlating with greater stability. Based on the predicted ΔG values, construct 2 appears to have a more stable mRNA secondary structure compared to construct 1, as the ΔG values for construct 2 are generally more negative, suggesting stronger base-pairing and a more thermodynamically favorable configuration. 

Additionally, the software generated energy dot plots for both constructs, providing a visual representation of the relative stability of different structural conformations by mapping the free energy of folding for each secondary structure. These energy dot plots further aid in evaluating the thermodynamic properties of the mRNA molecules and can be useful for identifying the most stable structures among the predicted alternatives.

### 4.9. Allergenicity and Antigenicity of Immunotoxins

Using the AlgPred server, we determined that both immunotoxins do not contain experimentally proven IgE epitopes and are not classified as allergens. This suggests that the constructs are unlikely to provoke allergic reactions in individuals, which is a crucial factor for their potential therapeutic use. The threshold for determining allergenicity was set at -0.4, and both construct 1 and construct 2 showed scores of -1.29 and -1.32, respectively, indicating that neither construct exceeded the threshold for potential allergenicity. These results further support the conclusion that both constructs do not exhibit significant allergenic potential. 

Additionally, the VaxiJen server, which predicts the antigenicity of proteins, indicated that both immunotoxins are probable antigens in tumor models. This suggests that, although the constructs are not allergens, they may still be recognized by the immune system as foreign antigens, potentially eliciting an immune response in the context of cancer immunotherapy. These findings are summarized in Appendix 4 in Supplementary File, which provides detailed information on the antigenicity predictions for both constructs.

## 5. Discussion

EphA2 is displayed on the cell surface, making it an ideal target for the design of immunotoxins. To develop an anti-EphA2 immunotoxin, we selected ricin as the toxin moiety. This protein toxin has already been used to generate other immunotoxins. Its small size is advantageous for cell internalization. Although ricin consists of two chains, A and B, only the A chain is required to kill cancer cells. The cytotoxic mechanism is related to the inhibition of the translation process inside the cells (23). 

Several factors should be considered when designing an immunotoxin (17). The critical considerations include vector size, expression, and solubility in the bacterial host, purification strategy, stability of mRNA and protein expressed from the construct, protein structure, binding ability of the immunotoxin to the cell surface, and its allergenic or antigenic potential. A smaller immunotoxin is preferable since a smaller vector is easier to construct using molecular cloning techniques (24), and the expressed protein is more likely to be soluble in the bacterial host (25). In this study, we aimed to generate two immunotoxins composed of subunit A of ricin and an antibody fragment (scFv) against the EphA2 receptor. Subunit A of ricin can be fused to the antibody at either its N- or C-terminal region. It has been shown that the orientation of the fused toxin relative to the antibody can significantly impact the efficiency of immunotoxins (26). 

Using bioinformatics tools to evaluate immunotoxin design and construction before experimental validation is highly beneficial. It is more cost-effective, saves time, and avoids ethical concerns related to the use of animal models for hypothesis testing. Experimental setups are expensive, and trial-and-error approaches are not advisable (16). Since the only difference between the two immunotoxins in this study is the orientation of the toxin relative to the antibody, we expect many physicochemical properties to be similar. Therefore, the aim of this study is to compare these two immunotoxins using bioinformatics approaches and identify the most promising candidate for combating breast cancer. 

The stability of the 3D structures of the designed immunotoxins is a crucial property. The structures obtained from 3D structure prediction tools demonstrated acceptable quality, indicating the accuracy of the predicted models. The low number of outlier amino acids within the generated Ramachandran plots further supports the accuracy of the predictions. However, the results suggest that the conformation of the protein expressed from construct 2 is slightly more favorable. Along with structural accuracy, the high binding potency of the designed immunotoxins to the target receptor is essential (27). Our analyses demonstrated that both immunotoxins are capable of binding to the EphA2 receptor with high affinity. However, construct 2 exhibited a stronger binding affinity to the EphA2 receptor and formed a greater number of stronger bonds with it. The MD simulation results further confirmed the stable binding between the designed immunotoxins and the EphA2 receptor. 

Based on these observations, it can be concluded that both designed immunotoxins could serve as suitable therapeutic candidates, but construct 2 exhibits more promising properties. The stability of the mRNA expressed from both constructs was assessed using the mFold software, and the results indicate sufficient stability. Based on thermodynamic properties, the mRNA expressed from construct 2 appears to be more stable. For the best model, the ΔG value for construct 1 was -613.69 kcal/mol, while for construct 2, it was -624.88 kcal/mol. Furthermore, the absence of experimentally proven IgE epitopes confirms the safety of the designed immunotoxins.

The obtained results in our study resemble the findings of the in silico sections of various studies. Ataee et al. conducted a bioinformatics study on the single-chain fragment variable of the rovalpituzumab antibody fused to granzyme B (Rova-GrB) and PltA of typhoid toxin (Rova-Typh) as immunotoxins. Their docking analysis demonstrated that the binding domain of the immunotoxins could bind to the N-terminal region of delta-like ligand 3 (DLL3). The bioinformatics analysis revealed that Rova-GrB and Rova-Typh possessed hydrophilic properties, their codon optimization parameters were within standard ranges, and their validation parameters were enhanced following immunotoxin refinement. They concluded that recombinant immunotoxins targeting DLL3 may serve as effective treatment options for small-cell lung cancer (SCLC) (28). 

In a similar study, Rezaie et al. assessed the characteristics of a proposed immunotoxin composed of an EphA2-specific scFv linked to PE38KDEL. They evaluated the binding potency, cytotoxic effects, apoptosis induction capacity, and internalization of the designed immunotoxin on an EphA2-overexpressing breast cancer cell line. Flow cytometry analysis demonstrated that, in contrast to the normal cell line (HEK-293) or the EphA2-low-expressing cell line (MCF-7), the immunotoxin could bind significantly (approximately 99%) to the EphA2-overexpressing breast cancer cell line (MDA-MB-231) at a low concentration (2.5 ng/μL). Moreover, significant cytotoxicity and apoptosis induction were observed in MDA-MB-231 cells at varying doses (10). 

In another study, Goleij et al. used recombinant DNA technology to fuse the HER2 monoclonal antibody (mAb) (Herceptin) to PE38, producing a novel recombinant immunotoxin. Their results showed that, in contrast to MCF-7 cells, SKBR-3 cells exhibited dose-dependent cytotoxicity when treated with this immunotoxin. The findings suggest that the immunotoxin may bind to HER2-positive breast cancer cells, undergo internalization, and induce apoptosis to eliminate the cancer cells (29). 

Furthermore, Mohammadi et al. (30) developed a unique recombinant anti-CD22 scFv.Bim fusion protein. They employed flow cytometry, microscopy, and the MTT assay to assess the binding capacity, cytotoxicity, and apoptotic activity of the purified recombinant protein against the CD22+ Raji cell line. Their analysis confirmed that anti-CD22 scFv.Bim exhibited apoptotic activity against Raji cells but not Jurkat cells. Additionally, in silico analyses demonstrated the good stereochemical quality of the 3D model and its molecular interactions with CD22. The researchers concluded that the pro-apoptotic peptide BIM could be effectively delivered to target cells by this novel recombinant anti-CD22 scFv.Bim fusion protein, making it a promising candidate for the treatment of B-cell malignancies. 

In light of these studies, it can be anticipated that the designed immunotoxins in our study may exert similar effects in eliminating appropriate cell lines and could potentially function as effective anti-cancer agents. 

### 5.1. Conclusions 

In this in silico study, we utilized bioinformatics analyses through various tools and web servers to evaluate the design and construction of two immunotoxins, construct 1 and construct 2. We assessed key parameters crucial for immunotoxin design, including protein structure, docking, MD simulation, mRNA stability, allergenicity, and antigenicity. Based on our analyses, construct 2 demonstrated superior properties, making it a more promising immunotoxin candidate. This construct exhibited better structural properties, higher binding affinity, and improved stability. We recommend that construct 2 undergo further experimental validation, including expression in a suitable bacterial host, purification, and in vitro testing on breast cancer cell lines to evaluate its therapeutic potential.

ijpr-24-1-151574-s001.pdf

## Data Availability

No dataset was used or generated during the conduct of this study.
