# Supplementary material for: Design and Characterization of Ricin Based Immunotoxins Against EPHA2 Receptor for Breast Cancer Therapy: An In-Silico Study
Source: Iran J Pharm Res. 2025 Feb 22;24(1):e151574. doi: 10.5812/ijpr-151574 (PMC12296653; doi:10.5812/ijpr-151574)
Supplement: ijpr-24-1-151574-s001.pdf [file ijpr-24-1-151574-s001.pdf]

Appendix 1. The quality scores for predicted secondary and tertiary structures of the Construct 1 and construct 2.

	**Construct 1**	**Construct 2**
**Secondary structure**	Alpha helix: 22.46%	Alpha helix: 23.92%
	Extended strand: 26.84%	Extended strand: 26.16%
	Random coil: 50.7%	Random coil: 49.91%
**Ramachandran plot**	Favored regions: 81.2%	Favored regions: 4.2%
	Disallowed regions: 82.2%	Disallowed regions: 1.6%
**QMEAN scores**	0.74	0.74
**ERRAT Overall Quality Factor**	86.5731	89.4212
**VERIFY 3D averaged 3D-1D score of >= 0.1**	73.84%	73.84%

Appendix 2. Physicochemical properties of the Construct 1 and construct 2.

	**Construct 1**	**Construct 2**
**Molecular weight**	62246.31 Da	63524.67 Da
**Pi**	9.07	8.97
**Extinction coefficient**	77615 M^{-1} cm^{-1}	77615 M^{-1} cm^{-1}
**Estimated half-life**	mammalian reticulocytes, *in vitro*: 30 hours	mammalian reticulocytes, *in vitro*: 30 hours
	yeast, *in vivo:* >20 hours	yeast, *in vivo:* >20 hours
	Escherichia coli, *in vivo:* >10 hours	Escherichia coli, *in vivo:* >10 hours
**Instability index**	39.33 meaning as a stable protein	38.65 meaning as a stable protein

Appendix 3. Docking results and binding affinity assessment of the Construct 1 and construct 2.

	**Construct 1 and EphA2**	**Construct 2 and EphA2**
**HADDOCK score**	914.6 +/- 47.0	926.1 +/- 47.1
**Prodigy binding affinity**	ΔG: -19.1 kcal/mol	ΔG: -20.9 kcal/mol
	Kd: 9.2e-15M	Kd: 4.6e-16M

Appendix 4. allergencity and antigenicity results of the Construct 1 and construct 2.

	**Construct 1**	**Construct 2**
**Allergencity**	-1.29	-1.32
**antigenicity**	probable antigen	probable antigens

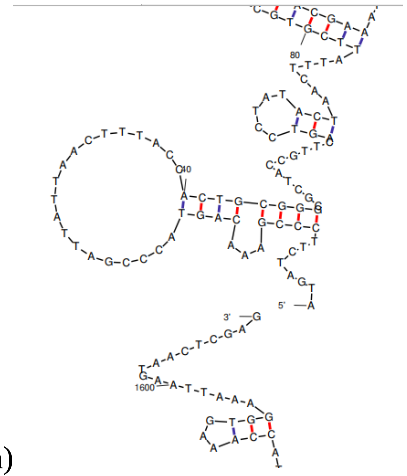

a)

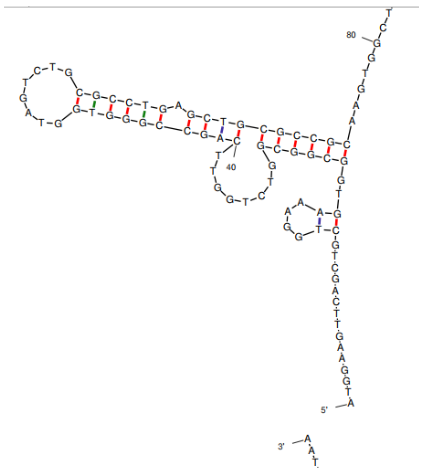

b)

Appendix 5. The energy plot of predicting mRNA structures of the Construct 1 (a) and construct 2 (b).
